# Supplementary material for: Perinatal and Neonatal Outcomes in Fetal Growth Restriction and Small for Gestational Age
Source: J Clin Med. 2022 May 12;11(10):2729. doi: 10.3390/jcm11102729 (PMC9143682; doi:10.3390/jcm11102729)
Supplement: Supplementary file 1 [file jcm-11-02729-s001.zip › jcm-1660862-supplementary.pdf]

Table S1. Delivery outcomes according to mode of delivery.

Features		AGA (N=655)	SGA (N=62)	Early-FGR (N=132)	late-FGR (N=57)
APGAR <7 1′					
	Caesarean Section	1 (0.2%)	2 (3.2%)	44 (33%) *** ### ^^^	3 (5.3%) ***
	Vaginal Delivery	4 (0.6%)	-	-	-
APGAR <7 5′					
	Caesarean Section	-	-	4 (3%)	-
	Vaginal Delivery	1 (0.2%)	-	-	-
pH < 7.10					
	Caesarean Section	2 (0.3%)	-	1 (0.8%)	2 (3.5%)
	Vaginal Delivery	16 (2.4%)	3 (4.8%)	-	-
EB					
	Caesarean Section	-1 (-1.15 – 0.6)	-1.8 (-2.3 – -1) **	-1.6 (-3.6 – -0.4) **	-2.1 (-4.3 – 1.8) ***
	Vaginal Delivery	-3.8 (-5.8 – -2)	-5.8 (-7.9 – -3.7) ** ## +	-5 (-6.1 – -2.9)	-3.8 (-5.2 – -2.6)
Lactate					
	Caesarean Section	1.6 (1.4-2.1)	2.4 (2-3.1) **	2.5 (2-3.3) **	2.2 (1.7-3.3) *
	Vaginal Delivery	4.1 (3.1-5.5)	4.9 (3.5-6.1)	4.5 (3.5-5.8)	4.2 (3.1-4.9)

Continuous variables are expressed as median (interquartile range). All the other features are expressed as categorical variables in frequencies and their percentage in the brackets. * *p* value < 0.05 vs AGA; ** *p* value < 0.01 vs AGA; *** *p* value < 0.001 vs AGA. ^^^ *p* value < 0.001 vs SGA. ## *p* value < 0.01 vs late-FGR; ### *p* value < 0.001 vs late-FGR. + *p* value < 0.05 vs early-FGR.
